# Sickle-trait hemoglobin reduces adhesion to both CD36 and EPCR by *Plasmodium falciparum*-infected erythrocytes

**DOI:** 10.1371/journal.ppat.1009659

**Published:** 2021-06-11

**Authors:** Jens E. V. Petersen, Joseph W. Saelens, Elizabeth Freedman, Louise Turner, Thomas Lavstsen, Rick M. Fairhurst, Mahamadou Diakité, Steve M. Taylor

**Affiliations:** 1 Division of Infectious Diseases, Duke University School of Medicine, Durham, North Carolina, United States of America; 2 Centre for Medical Parasitology, University of Copenhagen, Copenhagen, Denmark; 3 Laboratory of Malaria and Vector Research, National Institute of Allergy and Infectious Diseases, National Institutes of Health, Bethesda, Maryland, United States of America; 4 Malaria Research and Training Center, University of Sciences, Techniques, and Technologies of Bamako, Bamako, Mali; 5 Duke Global Health Institute, Duke University, Durham, North Carolina, United States of America; Seattle Children’s Research Institute, UNITED STATES

## Abstract

Sickle-trait hemoglobin protects against severe *Plasmodium falciparum* malaria. Severe malaria is governed in part by the expression of the *Plasmodium falciparum* Erythrocyte Membrane Protein 1 (PfEMP1) that are encoded by *var* genes, specifically those variants that bind Endothelial Protein C Receptor (EPCR). In this study, we investigate the effect of sickle-trait on parasite *var* gene expression and function *in vitro* and in field-collected parasites. We mapped *var* gene reads generated from RNA sequencing in parasite cultures in normal and sickle-cell trait blood throughout the asexual lifecycle. We investigated sickle-trait effect on PfEMP1 interactions with host receptors CD36 and EPCR using static adhesion assays and flow cytometry. *Var* expression *in vivo* was compared by assembling *var* domains sequenced from total RNA in parasites infecting Malian children with HbAA and HbAS. Sickle-trait did not alter the abundance or type of *var* gene transcripts *in vitro*, nor the abundance of overall transcripts or of *var* functional domains *in vivo*. In adhesion assays using recombinant host receptors, sickle-trait reduced adhesion by 73–86% to CD36 and 83% to EPCR. Similarly, sickle-trait reduced the surface expression of EPCR-binding PfEMP1. In conclusion, Sickle-cell trait does not directly affect *var* gene transcription but does reduce the surface expression and function of PfEMP1. This provides a direct mechanism for protection against severe malaria conferred by sickle-trait hemoglobin.

**Trial Registration:** ClinicalTrials.gov Identifier: NCT02645604.

## Author summary

Mutations that confer protection against severe malaria have evolved in humans, while malaria parasites have evolved extremely diverse *var* genes that encode proteins that enable the adhesion of infected red blood cells to host receptors to ensure parasite survival. In this study, we investigated the interplay between these two sides by examining the effect of the malaria protective sickle-cell trait hemoglobin mutations (HbAS) on the expression of parasite *var* genes, including those that cause severe malaria by mediating infected erythrocyte adhesion to host EPCR. We found that although *var* gene transcription was unaffected in vitro, HbAS reduced parasites ability to adhere to both CD36 and EPCR *in vitro*, by attenuating the expression of parasite proteins on the erythrocyte surface. Through RNA-sequencing we show that HbAS does not appear to cause any changes in overall *var* gene transcription, nor in the types of *var* genes expressed, and that children with HbAS mainly expressed *var* genes not associated with severe disease. Our results support a model in which HbAS confers protection from severe malaria by attenuating adhesion to host receptors.

## Introduction

Sickle hemoglobin (HbS) alleles are most prevalent in populations in areas with historically high *P*. *falciparum* malaria transmission. While HbS causes sickle cell disease in the homozygote state (HbSS), heterozygote carriers with sickle-cell trait (HbAS) have minimal sequelae. However, HbAS reduces the risk of severe, life-threatening *P*. *falciparum* malaria by over 90% [1].

These protective effects of HbAS against severe malaria are mediated by mechanisms that remain incompletely understood. Reduced parasite growth in HbAS red blood cells (RBCs) compared to normal HbAA RBCs in response to low oxygen tensions has been reported [2–4], while other studies did not find this effect [5,6]. Several lines of evidence support attenuation by HbAS of adhesion of infected red blood cells (iRBCs) to extracellular ligands, including to endothelial cells and specifically host receptors Chondroitin Sulfate A (CSA) and CD36 [7–10].

The binding of iRBCs to host receptors is mediated principally by a surface-expressed protein family, *P*. *falciparum* Erythrocyte Membrane Protein 1 (PfEMP1), which are large multidomain proteins encoded by highly sequence diverse *var* genes. Each haploid *P*. *falciparum* genome harbors around 60 unique *var* sequences grouped into A, B, C, and intermediate B/A, and B/C classes, based on chromosomal location, transcription direction and upstream untranslated region [11,12]. The exposed extracellular component of PfEMP1 is composed of multiple Duffy Binding-Like (DBL) and Cysteine-rich Interdomain Region (CIDR) domains, and a dichotomy of host-receptor specificity has evolved such that PfEMP1s with CIDRα domains bind either CD36 (CIDRα2–6 domains, found in group B, C, and B/C) or Endothelial Protein C Receptor (EPCR; CIDRα1 domains, found in group A and B/A PfEMP1s). Within each parasite, *var* gene transcription is mutually exclusive, and switching between the actively transcribed *var* gene serves as a mechanism of immune evasion [13], and this clonal antigenic variation also determines the iRBC’s host receptor interaction. These host-receptor interactions are associated with malaria pathogenesis and clinical outcomes. Placental malaria is associated with the expression of the CSA-binding PfEMP1 known as VAR2CSA [14]. The *P*. *falciparum* genome is primarily comprised of *var* genes encoding CD36-binding PfEMP1s and their expression is not associated with severe disease. However, severe disease in children and adults is associated with the expression of the less-abundant PfEMP1s containing the EPCR-binding CIDRα1 [15–18].

Altered PfEMP1s transport and presentation on the RBC surface along with aberrant actin polymerization has been shown for HbAS iRBCs [8,19]. However, it remains unclear how HbAS affects the iRBC’s host receptor specificity and ability to cytoadhere to one of the major host interaction partners, EPCR, and how HbAS impacts *var* gene transcription and switching between *var* genes.

In this study, we investigated the effects of sickle-trait hemoglobin on overall *var* transcription and on PfEMP1 expression and function. To do so, we compared *var* transcriptomes of parasites in HbAA and HbAS both during *in vitro* cultivation and in Malian children with uncomplicated malaria, and we compared *in vitro* the effect of HbAS on EPCR and CD36 binding. Given that clinical severity of malaria is linked to both HbAS and to *var* transcription, we hypothesized that, compared to HbAA RBCs, HbAS would reduce the binding of iRBCs to EPCR by attenuating the expression of *var* transcripts and their PfEMP1 products that bind EPCR.

## Results

### Sickle trait does not alter *var* gene transcription *in vitro*

To comprehensively investigate the effect of sickle trait on *var* gene expression, we used RNA sequencing to compare the full *var* gene transcriptional repertoire for 3D7 parasites growing in HbAA and HbAS RBCs. We sampled tightly-synchronized 3D7 parasites growing in parallel in HbAA and HbAS RBCs every 3 hours over a single 48-hour life-cycle and quantified reads mapping to *var* genes for each timepoint and hemoglobin type.

**Fig 1:**
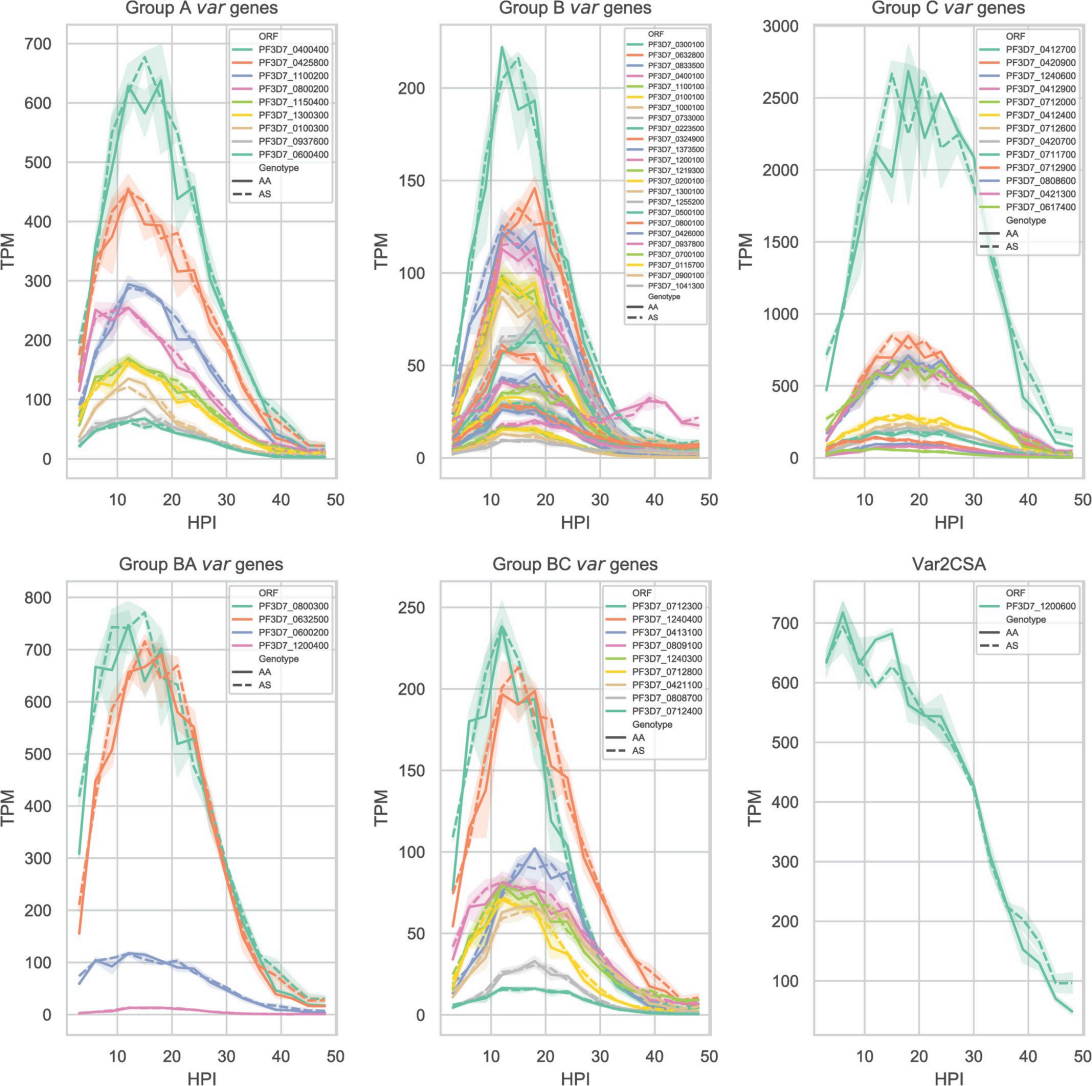
3D7 *var* gene expression across the asexual stage in blood from HbAA and HbAS donors. *Var* gene transcription relative to total RNA transcripts per million (TPM), every three hours post invasion (HPI) for 48 hours. *Var* genes are grouped according to their UPS classification, and individual *var* genes are represented by different colors according to their open reading frame (ORF). Solid lines represent average transcription of two HbAA donors, and dashed lines the average transcription of two HbAS cultures, and shaded areas are the min and max replicate value. Note difference in y-axis scales between panels.

Parasites growing in HbAA and HbAS had a similar parasitemia at around 3%, and matured equally and progressed normally throughout the time-course (Joseph Saelens, manuscript in preparation). In both HbAA and HbAS RBCs, we observed measurable expression of all major *var* gene groups: A, B, C, B/A, B/C, and pregnancy-associated *var2csa* (Fig 1). As expected, *var* transcripts generally peaked between 12 and 21 hours post invasion (HPI), before declining as the parasites matured into schizont stages at around 42 HPI. One notable exception, consistent with prior reports [20], was the earlier expression of *var2csa* during the early ring stages. Although the 3D7 parasite cultures had not been subjected to any phenotype selection favoring specific *var* gene expression, the group C *var* gene PF3D7_0412700 was expressed more than 3-fold higher than any other *var* gene.

Comparatively, *var* gene expression profiles across the 48 hours post invasion were remarkably similar between parasite cultures in HbAA and HbAS. Compared to parasites in HbAA RBCs, we did not observe in parasites growing in HbAS RBCs any differences in either timing or amplitude of transcription for individual *var* transcripts or for *var* gene subgroups.

### Sickle-cell trait impairs adhesion to CD36 and EPCR

In light of this unaltered *var* gene transcription in HbAS and of prior reports of reduced surface expression of PfEMP1 and adhesion of infected HbAS RBCs to endothelial cells and key host receptors [7–9,19], we investigated whether our 3D7 parasites in HbAS RBCs had attenuated adherence to host receptors.

**Fig 2:**
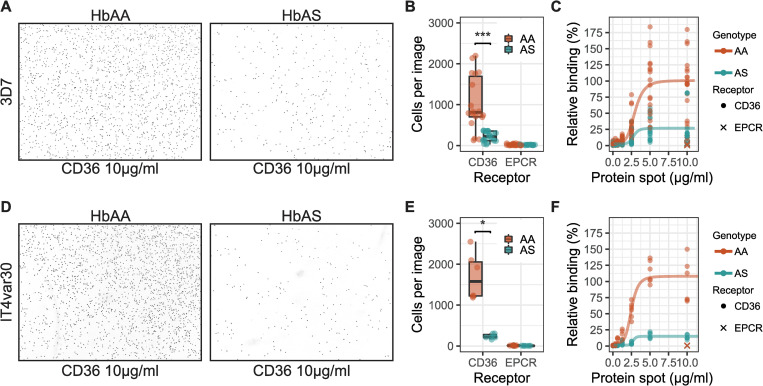
Sickle-cell traits effect on parasite adhesion to CD36. **A)** HbAA or HbAS RBCs infected with 3D7 reference parasite strain adhering to spots of recombinant CD36 on a petri-dish. **B)** Quantification of the adhesion of 3D7-infected HbAA and HbAS erythrocytes to 10 μg/ml CD36 and EPCR spots. Each condition was done as 2 protein spots on separate petri-dishes, each imaged 3 times. The assay was done 3 independent times (n = 3). Wilcoxon’s signed rank test was used to evaluate statistical significance (*** p-value = 0.0001). **C)** Relative adhesion across protein spots of different concentrations normalized to the mean adhesion to CD36 at 10 μg/ml. Four parameter logistic curve was fitted to the normalized data for HbAA and HbAS. **D)** HbAA or HbAS RBCs infected with IT4 strain parasites expressing IT4var30-PfEMP1 (IT4var30) adhering to spots of recombinant CD36 on a petri-dish. **E)** Quantification of the adhesion of It4var30-infected HbAA and HbAS erythrocytes to 10 μg/ml CD36 and EPCR spots. Each condition was done as 2 protein spots on separate petri-dishes, each imaged 3 times. Wilcoxon’s signed rank test was used to evaluate statistical significance (*p-value = 0.03). **F)** Relative adhesion across protein spots of different concentrations normalized to the mean adhesion to CD36 at 10 μg/ml. Four parameter logistic curve was fitted to the normalized data for HbAA and HbAS (n = 1).

We observed high basal expression in 3D7 of PF3D7_0412700 (Fig 1), which binds CD36 [21]. Therefore, we first compared adhesion to recombinant CD36 using synchronized trophozoite-stage 3D7 parasites in HbAA and HbAS RBCs (Fig 2A). In technical and biological replicates of both RBCs and protein preparations, the mean number of 3D7 parasites per field of view bound to CD36 at 10 μg/ml was significantly higher in HbAA RBCs (1028; 95% CI: 698–1359) than in HbAS RBCs (217; 95% CI; 154–279) (P = 0.0001 by Wilcoxon signed-rank test) (Fig 2B). The adhesion was CD36 concentration-dependent, but reduced adhesion for infected HbAS RBCs was observed across a range of CD36 concentrations (Fig 2C). When fitting a four-parameter log-logistic model to the adhesion of infected HbAA and HbAS RBCS across CD36 spot concentrations, the estimated 50% adhesion for parasites was similar in HbAA (2.9 μg/ml) and HbAS (2.8 μg/ml). In these models, the maximum adhesion of HbAA was 100.8 (6.1 Std. error) while HbAS was 26.8 (2.5 Std. error), corresponding to 73% lower maximum adhesion to CD36 for HbAS iRBCs.

To test if this reduced binding to CD36 for HbAS iRBCs was unique to the unselected 3D7 parasite line, we examined the adhesion of a *P*. *falciparum* strain IT4 that was selected to express the CD36-binding PfEMP1 variant IT4var30 (hereafter IT4var30, [22,23]). In this selected line, the mean number of iRBCs per field of view bound to 10 μg/ml CD36 spots was 1702 (95% CI: 1099–2304) in HbAA RBCs and significantly reduced to 234 (95% CI: 174–294) in HbAS RBCs (P = 0.03, Wilcoxon signed-rank test) (Fig 2D and 2E). Similar to 3D7, adhesion of IT4var30 to CD36 was concentration dependent but adhesion of HbAS iRBCs was reduced across a range of CD36 spot concentrations (Fig 2F). When fitting a four-parameter log-logistic model to IT4var30 adhesion to CD36 in HbAA or HbAS, 50% adhesion was estimated to be similar for HbAA (2.5 μg/ml) and HbAS (2.7 μg/ml). In these models, the maximum adhesion of HbAA iRBCs was 108% (5.5 Std. error), while HbAS was 15% (0.8 Std. error), corresponding to 86% lower maximum adhesion to CD36 for HbAS iRBCs.

**Fig 3:**
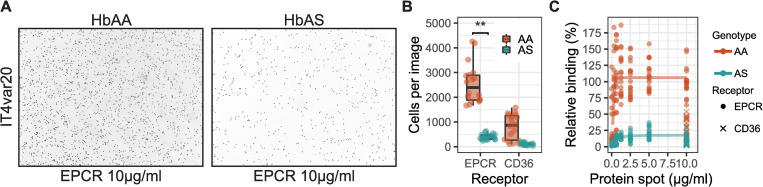
Sickle-trait hemoglobin effects on erythrocyte adhesion to EPCR. **A)** Erythrocytes infected with IT4 parasite-strain expressing IT4var20 adhering to spots of recombinant EPCR on a petri-dish. **B)** Quantification of IT4var20-infected normal (HbAA) erythrocytes and sickle-cell trait (HbAS) erythrocytes adhesion to 10 μg/ml EPCR and CD36 spots. Each condition was done as 2 protein spots on separate petri-dishes, each imaged 3 times. The assay was done 3 independent times (n = 3). Wilcoxon’s signed rank test was used to evaluate statistical significance (** p-value = 0.0002). **C)** Relative adhesion across protein spots at different concentrations normalized to the mean adhesion to EPCR at 10 μg/ml. Four parameter logistic curve was fitted to the normalized data for HbAA and HbAS.

Severe malaria in children is strongly associated with the expression of PfEMP1 variants predicted to bind EPCR [17,24], therefore we next tested if HbAS affected the adhesion of iRBCs to EPCR. For these experiments, we used IT4 parasites that were selected to express the EPCR-binding PfEMP1 IT4var20 (hereafter IT4var20, [17]) and assayed the adhesion to recombinant EPCR. The mean number of adherent IT4var20-infected RBCs to EPCR was 2549 in HbAA (95% CI: 2149–2950) and was significantly reduced to 393 (95% CI: 335–451) in HbAS (P = 0.0002 by Wilcoxon signed-rank test) (Fig 3A and 3B). Adhesion to EPCR by IT4var20 was concentration dependent, and when fitting a four-parameter log-logistic model (Fig 3C) 50% adhesion was estimated at 0.3 μg/ml and 0.5 μg/ml EPCR spots for HbAA and HbAS, respectively. In these models, maximum adhesion for IT4var20 in HbAA was 106.1 (4.3 Std. error) and for HbAS was 17.8 (1.2 Std. error), corresponding to a 83% lower maximum adhesion to EPCR for iRBCs with HbAS.

### Sickle-cell trait reduces surface PfEMP1 surface expression

**Fig 4:**
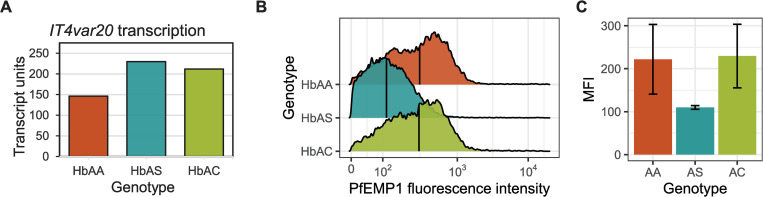
Effects of sickle-trait hemoglobin on transcript and surface protein expression of IT4var20 PfEMP1. **A)** Quantitative PCR of *IT4var20* transcription in HbAA, HbAS, and HbAC converted to Transcript units relative to *seryl-tRNA synthetase* housekeeping gene (n = 1). **B)** Histogram of IT4var20 PfEMP1 expression from a representative flow cytometry experiment. The PfEMP1 fluorescence intensity was measured from infected HbAA, HbAS, and HbAC RBCs, stained with monoclonal IgG antibody targeting the CIDRα of IT4var20 and APC-conjugated anti-mouse IgG. The median flourescence is marked with a vertical line. **C)** Summary of the median fluorescence intensity (MFI) from separate flow cytometry experiments (n = 3). Error bars show the standard deviation.

We next tested if this binding phenotype was supported by aberrant *var* gene expression phenotypes in the IT4var20 parasite line that binds EPCR in HbAS RBCs. We first used qPCR to assess expression of *IT4var20* in ring-stage IT4var20 parasites in parallel HbAA, HbAS, and RBCs carrying a different hemoglobin mutation (HbAC) by measuring transcript units (Tu), a measure of transcript abundance relative to house-keeping gene *seryl-tRNA synthetase* [18]. Relative to iRBCs with HbAA, transcript levels of IT4var20 were equally high in iRBCs with HbAS or HbAC (Fig 4A). We next assessed the surface PfEMP1 expression by flow cytometry for trophozoite stage IT4var20 using monoclonal antibodies targeting the EPCR-binding site of IT4var20. Compared to iRBCs with HbAA, IT4var20 surface levels were unaffected in HbAC, but lower in HbAS iRBCs (Fig 4B and 4C). Collectively, our *in vitro* data indicate that HbAS allows normal *var* transcript expression but reduces the PfEMP1 surface presentation and hence functional adherence to endothelial receptors including CD36 and EPCR.

### *Var* gene transcription in sickle-trait patients

**Fig 5:**
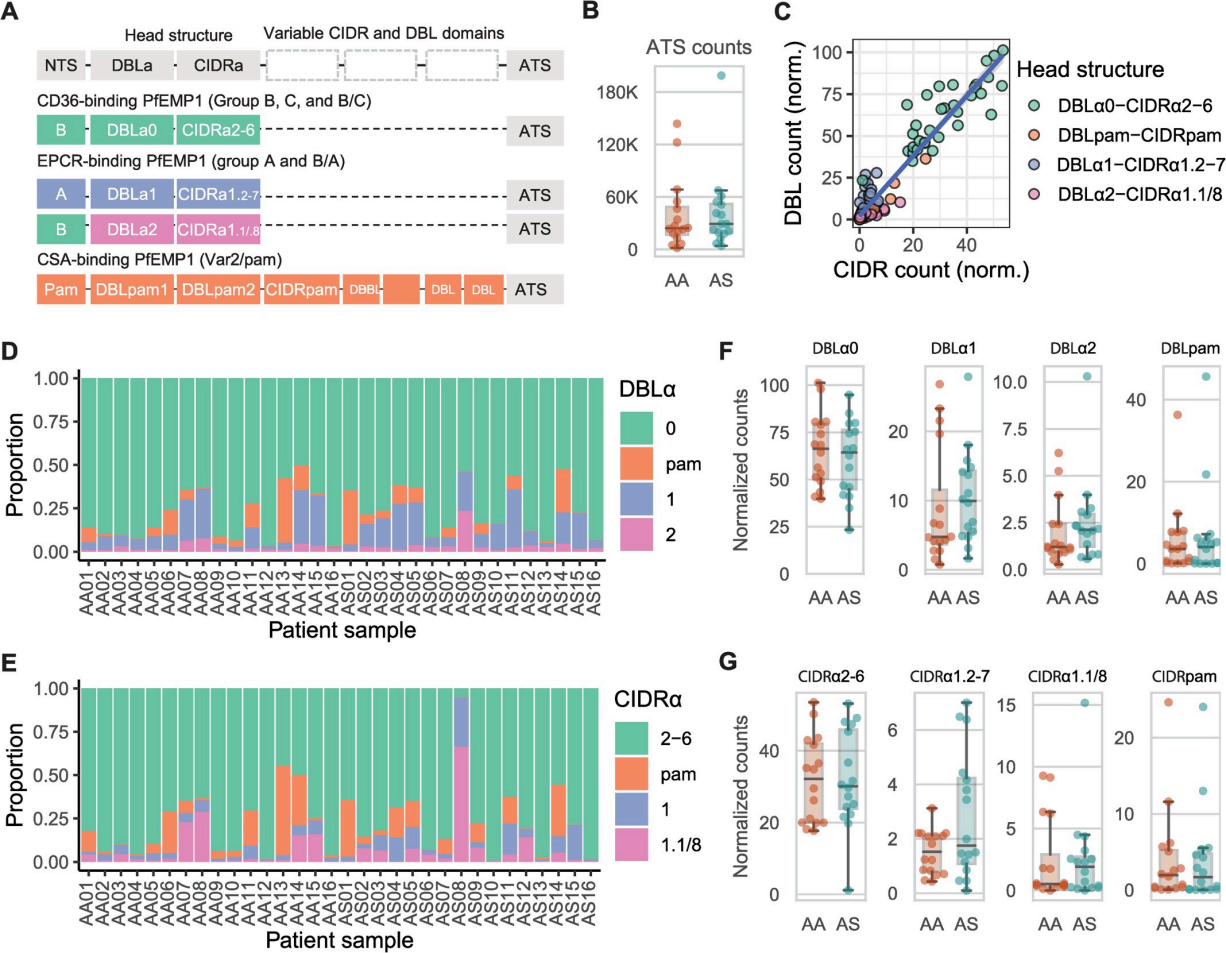
*Var* domain expression in HbAA and HbAS children in Mali. **A)** PfEMP1 domain schematic showing the domain composition of the receptor-binding headstructures of CD36-binding PfEMP1s belonging to group B, C, and B/C; EPCR-binding PfEMP1s belonging to Group A, and B/A; and CSA-binding PfEMP1s, Var2. **B)** Total read counts for the acidic-terminal segment (ATS) sequences for each of the 32 patient samples. Boxplots show the distribution according to hemoglobin genotype, and the individual patients samples are represented by dots. **C)** Normalized read counts for DBL and CIDR domains according to head structure within each patient. Read counts were normalized to ATS domain read counts for each patient. A linear model trendline is marked in blue. **D)** Proportion of read counts for DBLα0, DBLα1, DBLα2, and DBLpam1 domains normalized to ATS read counts in individual patient samples. Colors correspond to head structure type. **E)** Proportion of ATS-normalized readcounts for CIDRα2–6, CIDRα1, and CIDRpam domains. **F)** Normalized read counts of DBLα0, DBLα1, DBLα2, and DBLpam1 domains in children with HbAA and **G)** Normalized read counts for CIDRα2–6, CIDRα1, and CIDRpam domains in children with HbAA Boxplots show distribution according to hemoglobin genotype, and the individual patient samples are represented by dots. Boxplots show median, interquartile range (IQR), and whiskers show 1.5 times the IQR.

We next tested the *in vivo* effect of HbAS on *var* gene transcription, by comparing *var* transcript profiles between 16 matched pairs of Malian children with uncomplicated malaria and either HbAS or HbAA (Table 1). Using reads obtained from a separate comprehensive RNA-sequencing comparison, (*manuscript in preparation*), we defined a subset of reads that failed to map to both non-*var* 3D7 genes and human genes and mapped these to a library of 2,476 annotated *var* gene domains from 395 *var* genes [11,15]. This allowed us to measure within each infection the overall *var* transcript abundance by assessing the levels of shared conserved domains and the relative expression of *var* genes encoding specific subtypes of N-terminal PfEMP1 domains (Fig 5A) while mitigating the challenge of mapping the highly diverse *var* reads.

**Table 1:** Summary of matched HbAA and HbAS study participant characteristics. Blood samples from 32 children with uncomplicated *P*. *falciparum* malaria in Mali: 16 samples from children with hemoglobin AS genotype (AS) and 16 matched samples from children with hemoglobin AA genotype (AA). SD, standard deviation.

	AA (N = 16)	AS (N = 16)	Total (N = 32)	p value
**Gender**				1.000^1^
Female	6 (37.5%)	6 (37.5%)	12 (37.5%)	
Male	10 (62.5%)	10 (62.5%)	20 (62.5%)	
**Age (Years)**				0.083^2^
Mean (SD)	8 (3)	9 (3)	8 (3)	
Range	3–13	3–14	3–14	
**Parasite density (μL)**				0.117^3^
Mean (SD)	30230 (16693)	19842 (14590)	25036 (16300)	
Range	9125–66100	4950–52650	4950–66100	
**Blood type**				0.381^1^
A	6 (37.5%)	2 (12.5%)	8 (25.0%)	
AB	2 (12.5%)	4 (25.0%)	6 (18.8%)	
B	4 (25.0%)	6 (37.5%)	10 (31.2%)	
O	4 (25.0%)	4 (25.0%)	8 (25.0%)	

1. Pearson’s Chi-squared test

2. Paired t-test

3. Wilcoxon signed rank exact test

As an index of overall *var* gene expression, we compared the abundance of reads mapping to the conserved C-terminal acidic-terminal segment (ATS) that is found in all PfEMP1s. There was no difference in mean ATS domain read counts between patients with normal (mean = 39,590, Std. deviation = 39,556) and sickle-cell trait (mean = 42,235, Std. deviation = 44,505) hemoglobin (P = 0.79 by Kruskal-Wallis test) (Fig 5B), suggesting that the overall abundance of *var* gene transcripts was similar between the children with HbAS and HbAA.

Indexed to these ATS domain counts, we then analyzed expression of N-terminal *var* gene head structure domains which predict or directly bind the major PfEMP1 host binding partners: CD36, EPCR, or CSA (Aggregated N-terminal domain raw read counts summarized in S1 Table). Because specific DBLα and CIDRα domain types are found together in PfEMP1’s head structure [11,25], we analyzed the correlation of DBLα and CIDRα domain expression stratified by head structure type. The expression (normalized read counts) of DBL and CIDR domains of the same head structure were highly correlated across the patient samples (R^2^ = 0.9, Fig 5C). Thus, DBLα0 and CIDRα2–6 domains of CD36-binding head structures were highly correlated; DBLα1/2 and CIDRα1 domains of EPCR-binding head structures were highly correlated; and DBLpam and CIDRpam from *var2csa* domains were highly correlated. The high correlation of transcript levels of co-occurring DBL and CIDR domains, suggests that the approach accurately quantifies the transcript levels of the individual domains.

When analyzed by functional domains, the dominantly transcribed DBLα domain was DBLα0 (Fig 5D) and that of CIDRα was CIDRα2–6 (Fig 5E), indicating that parasites in children with both HbAA and HbAS predominantly transcribe CD36-binding PfEMP1s (Fig 5D and 5E).

We next compared read counts between groups of specific DBLα domains found in 95% of *var* genes, defined by their shared amino acid identity [11]. DBLα0 domains were transcribed at similar levels between children with HbAA (mean = 65.6) and HbAS (mean = 60.9, P = 0.5 by Welch’s t-test) (Fig 5F). Normalized read counts for DBLα1 were also similar in HbAA (mean = 8.9) and HbAS (mean = 10.5, P = 0.6 by Welch’s t-test), as well as for DBLα2 in HbAA (mean = 1.9) and HbAS (mean = 2.5, P = 0.4 by Welch’s t-test).

For the CD36-binding CIDRα2–6 domains, normalized read counts were similar between children with HbAA (mean = 31.9) and with HbAS (mean = 32, P = 0.9 by Welch’s t-test) (Fig 5G). Similarly, the EPCR-binding CIDRα1 domains did not differ between children with HbAA (mean = 1.9) and HbAS (mean = 2.67, P = 0.3 by Welch’s t-test). Additionally, there were no differences between HbAA and HbAS children in the transcription of PfEMP1 domains not belonging to the head structure (S1 Fig).

## Discussion

We investigated the effects of HbAS on *var* gene expression *in vitro* and *in vivo*, on PfEMP1 expression on the iRBC surface, and on PfEMP1-mediated adhesion of *P*. *falciparum*-infected RBCs to host endothelial receptors. HbAS did not affect *var* gene expression across PfEMP1 types either in reference parasite cultures or in parasite samples directly from children in Mali with uncomplicated falciparum malaria. Despite this absence of an effect on *var* gene transcription, we show for the first time that HbAS reduces the surface expression of a PfEMP1 variant that mediates adhesion of iRBCs to EPCR and that this produces a functional deficit in adhesion to recombinant EPCR. Given that severe malaria results in part owing to the binding of iRBCs to EPCR *in vivo* and that sickle-trait confers near-complete protection against severe malaria, these results suggest that sickle-trait confers direct protection from severe malaria by attenuating the ability of infected RBCs to interact with EPCR.

HbAS reduced expression of EPCR-binding PfEMP1s on the surface of the iRBC and the binding of iRBCs to EPCR by 83%. Among parasitologic factors, severe malaria is strongly associated both with higher parasite biomass and with the specific expression of *var* genes encoding PfEMP1s that are predicted to bind EPCR [24]. Under normal conditions, EPCR functions as a scaffold on which protein C is activated to enable its participation in the anticoagulant pathway, and it is important for maintaining vascular and tissue integrity in multiple organs including the brain, lung and kidneys [26]. EPCR was identified as a binding partner for parasite proteins and a critical mediator of iRBC sequestration in severe malaria syndromes [17,18,27,28]; specifically, adherence of iRBCs to EPCR is mediated by PfEMP1 variants that harbor CIDRα1 domains. These domains bind EPCR with extremely low off-rates, and this interaction blocks the normal functions of EPCR in endothelial activation and maintaining barrier integrity [29–31], which promotes coagulopathy, inflammation and vascular leakage, all of which contributes to severe malaria such as cerebral malaria [32–34]. Thus, our observation that HbAS significantly reduces expression of EPCR-binding PfEMP1 as well as adhesion of iRBCs to EPCR provides a direct mechanism by which HbAS confers protection from severe malaria.

Similarly, adhesion to CD36 was reduced by HbAS RBCs infected with CD36-binding parasites that either were (IT4var30) or were not (3D7) selected for the adhesion phenotype. CD36 is expressed on both endothelial and immune cells, and its functions involves mediating lipid uptake, recognizing phospholipids and lipoproteins and phagocytosis of pathogens [35]. It is the most common host interaction partner for PfEMP1s: 80% of the *var* gene repertoire contains CD36-binding CIDRα2–6 domains, while only 10% of *var* genes contain EPCR-binding CIDRα1 domains. [11,25]. *Var* genes encoding CD36-binding PfEMP1s have been shown to dominate in uncomplicated malaria in children [15]. We observed in HbAA RBCs a higher adhesion to CD36 for IT4var30 compared to the unselected 3D7 parasites. This difference may be due to differences between the strains and the specific expressed PfEMP1, routine selections for adhesion phenotype affects PfEMP1 surface expression and proper knob formation [36–38]. Despite this baseline difference between parasite strains, adhesion of IT4var30 and 3D7 to CD36 spots had comparable dose responses across CD36 concentrations as well as reductions in binding in HbAS RBCs, likely reflecting the similar binding kinetics between the PfEMP1s expressed on the two strains and CD36. Collectively, these results provide direct functional evidence that HbAS reduces iRBC adhesion to the major PfEMP1 interaction partners that mediate uncomplicated malaria and severe malaria syndromes. Our findings do not exclude other potential ways HbAS can confer protection against severe malaria, and whether the protection is contingent upon abrogating the specific PfEMP1 interaction with EPCR, or PfEMP1 interactions in general, remains unresolved. As HbAS does appear to indiscriminately lower iRBC adhesion, the protective effects of HbAS could be due to overall lower adhesion that would facilitate faster clearing of iRBCs, resulting in lower parasite burden.

Interestingly, despite these functional defects in PfEMP1 surface expression and in adhesion, HbAS did not alter *var* gene expression. We did not observe shifts in amplitude, timing or switching between the individual *var* genes, either during *in vitro* cultivation or *in vivo* among matched children with HbAA or HbAS and uncomplicated malaria. Although our *in vitro* sequence data was not done with parasites specifically selected to express an EPCR-binding PfEMP1 and the expression was largely dominated by *var* genes encoding CD36-binding PfEMP1, we were able to quantify *var* gene expression of both *var2csa* and *var* genes predicted to encode EPCR-binding PfEMP1s. iRBCs in both HbAA and HbAS children with uncomplicated malaria predominantly expressed domains belonging CD36-binding group B and C *var* genes. This abundance of *var* transcripts encoding CD36-binding PfEMP1 is consistent with previous reports of patients with uncomplicated malaria [15]. In addition, there was no evidence for shift in associated host receptor preference or a change in *var* gene transcript levels associated with HbAS genotype, in that parasites had similar overall *var* gene expression levels and frequencies of transcripts of *var* gene domain types (belonging to PfEMP1s with either CD36, EPCR or CSA as their primary host receptor). Because HbAS reduces the capacity of EPCR-binding PfEMP1s to cause severe malaria, we might expect in uncomplicated malaria patients with HbAS a corresponding increase in the frequency of EPCR-binding PfEMP1s. Although our 16 pairs of HbAA and HbAS samples might of insufficient size to identify smaller shifts in *var* gene expression preferences, the HbAS samples did not show signs of major *var* gene expression divergence. Despite some heterogeneity between individual patients, there was not a statistically significant difference between uncomplicated malaria patients with HbAA or HbAS in the number of *var* gene transcripts belonging to PfEMP1s that are predicted to bind EPCR.

The twin observations that HbAS reduces PfEMP1 surface expression but does not disrupt the expression of transcripts encoding PfEMP1 suggests that a critical effect of HbAS is post-transcriptional. These could include defects in PfEMP1 translation, aberrant PfEMP1 transport to the erythrocyte surface, or increased degradation of either *var* transcripts or PfEMP1 proteins. Disrupted transport of PfEMP1 to the RBC surface is supported by prior studies which observed aberrant movement and structure in HbAS RBCs of Maurer’s clefts, which serve to shuttle parasite-derived proteins to the RBC surface [39], and infected HbAS RBCs have overall changes in expression of Mauer’s cleft genes (Joseph Saelens, *manuscript in preparation*). Additionally, aberrant actin polymerization and altered of surface knob structures on HbAS iRBCs have been associated with loss of adhesion, and reduced endothelial activation in HbAS RBCs *in vitro* [8,10,19].

We used a novel approach to overcome the great sequence diversity *var* genes in freshly isolated parasites. To do so, we leveraged new understanding of a relevant functional typology of var genes and focused on *var* gene domains with defined subtypes and known conserved domain compositions [11]. Cataloging *var* transcripts in natural infections is a challenge owing to the large sequence diversity which makes mapping sequence reads to reference *var* gene templates nonsensical. Prior studies have examined *var* gene expression on an overall level through analysis of conserved DBLα-tag sequences or with primers designed to amplify specific *var* gene domains [18,40]. RNA sequencing of *var* genes has previously been investigated with de-novo assembly and mapping reads to PfEMP1 domains [41]. We mapped short read fragments of 15 bases from RNA sequencing runs to a library of 2476 annotated *var* gene domains from multiple parasite isolates [11,15]. From this we were able to quantify expression of domains belonging to these different functional subtypes within the individual patients. The validity of this approach was supported by the observation that the relative expression of DBLα domains were highly correlated with expression of CIDRαs belonging to the same head-structure (co-occurring DBLα and CIDRα domains), indicating that the approach was not biased towards specific DBL or CIDR domains. Additionally, the N-terminal segments (NTS) transcribed in each individual matched the head-structures (S2 Fig). Potential biases towards specific *var* gene types, would be equal for both HbAA and HbAS samples, but the frequencies of PfEMP1 domain types found in our uncomplicated malaria cases were in accordance with previous studies, where DBLα tag PCR sequencing, and long-range PCR on genomic DNA was used to assemble and annotation of the identified *var* genes to identify the highest transcribed *var* genes [15]. The approach presented here can be adapted more broadly to reliably capture transcript profiles of functionally relevant *var* domains in clinical subtypes of malaria from RNA-sequencing data.

Our study had several limitations. Functional studies of the effect of HbAS on *P*. *falciparum* are limited to *in vitro* studies, as there are no appropriate animal models in which we can study HbAS effect on parasite growth and iRBC adhesion. Owing to this, we examined *in vivo* transcripts with clear functional correlations as a proxy. Our static adhesion assays do not precisely replicate the local environment in a capillary, owing to the host receptors not being endogenously expressed and presented on the surface of an endothelial cell, and the lack of shear stress imposed by the blood flow. Our EPCR-binding IT4var20 parasite culture did not purely express IT4var20, as evidenced by a subpopulation that was negative in flow cytometry (Fig 4B), and despite repeated selecting for IT4var20 expression with antibody and recombinant EPCR. This sub-population could explain why we observe residual adhesion to CD36 for this parasite line. Lastly, our *in vivo var* gene transcript analysis was limited by not having genomic DNA from the patient samples sequenced which could have aided in de-novo assembly of the *var* genes present in each patient.

In conclusion, HbAS did not impact *var* gene transcription throughout the intra-erythrocytic lifecycle but did reduce both surface expression of PfEMP1 as well as general PfEMP1-mediated binding to host receptors. Importantly, we observed an 83% reduction in binding of iRBCs to the host receptor EPCR, which is a host-parasite interaction that is central to the pathogenesis of severe malaria. Our findings provide evidence of a specific mechanism underlying the protection against severe malaria conferred by sickle-trait hemoglobin.

## Methods

### Ethics statement

The sample collection was reviewed and approved by the Institutional Review Board of the University of Sciences, Techniques, and Technologies of Bamako (IRB00001983), and Duke University Hospital (IRB # Pro00007816 and Pro00101991). Written consent was obtained from all study participants, their parents or their legal guardians.

### Erythrocyte collection for parasite cultivation

HbAA and HbAS RBCs were obtained from healthy donors (IRB # Pro00007816 and Pro00101991) and beta-globin type was confirmed by PCR amplification and Sanger sequencing. For each experiment, HbAA and HbAS RBCs were collected within 1 day of each other to minimize differences in erythrocyte age. RBCs of each type were used for up to two weeks after donation.

### Parasite culture

*P*. *falciparum* parasites were grown in Albumax complete medium (1640 RPMI supplemented with Albumax II, L-Glutamine, Hypoxanthine, and Gentamicin) according to established protocols [42] and maintained in a 1% O_2_, 5% CO_2_ in N_2_ gas mixture at 37°C. Cultures were synchronized with 5% sorbitol for ring stage synchronization, and 70% Percoll was used for purifying trophozoite and schizont stages. 3D7 was acquired from BEI Resources, NIAID, NIH (MRA-102, contributed by Daniel J. Carucci). IT4var30 and IT4var20 were generated as previously described [17,23], and *var* gene expression was confirmed using qPCR. PfEMP1 expression was maintained by routine selections with specific PfEMP1-antibodies, and either recombinant EPCR (Sino biological) or recombinant CD36 (Abcam) as follows: Antibody or recombinant protein was adsorbed to the bottom of a tissue culture flasks overnight at 4°C. Flasks were washed with Albumax complete media, and late-stage parasite cultures were added and incubated for 1 hour under gentle nutation at 37°C. Unbound RBCs were washed away and fresh blood was added to the flasks.

### Time course

For *in vitro* time-series experiments, tightly synchronized 3D7 schizonts were isolated by Percoll centrifugation. The schizonts were inoculated into HbAA and HbAS RBCs; after a 3-hour incubation to enable merozoite egress, parasite cultures were treated with sorbitol to remove unruptured schizonts. Every third hour samples were stored in Trizol (Invitrogen) for RNA isolation and prepared as Giemsa-stained smears for assessment of parasite maturation using light microscopy.

### Field study sample collection

Fresh *P*. *falciparum* parasites were collected in an observational study of malaria in children in Kenieroba, Mali (ClinicalTrials.gov Identifier: NCT02645604). Written consent was obtained from parents or guardian of participating children. In this area, *P*. *falciparum* is transmitted intensely each rainy season, innate red cell variants are common, and HbAS reduces the risk of uncomplicated malaria by 34% [43]. From children presenting with uncomplicated *P*. *falciparum* malaria, venous blood was collected, passed through cellulose columns [44], and up to 2mL of the flow-through was stored in RNAprotect (Qiagen) in cryovials. We selected all available samples from children with HbAS, and matched each of these to a sample from a child with HbAA on: month of episode, parasite density, ethnic background, and, if possible, ABO blood type.

### RNA extraction

Trizol samples were mixed with chloroform and the RNA-containing aqueous phase was collected following centrifugation. RNA was extracted using RNeasy kit (Qiagen), with on column DNase treatment. RNA was eluted in 30 μl RNase free water, and concentrations were measured by Qubit High Sensitivity RNA Assay (Thermo Fisher Scientific). RNA was stored at -80°C. Samples from Mali patients were treated with 0.75mL of TRIzol LS Reagent (Thermo Fisher) per 0.25mL of sample volume, and RNA was subsequently extracted and stored as described above for *in vitro* time series samples.

### Quantitative PCR

From RNA samples, cDNA was synthesized using AffinityScript RT kit (Agilent) according to manufacturer’s protocol. cDNA concentrations were measured fluorometrically using Qubit (Thermo Fisher Scientific). Quantitative PCR was performed with iTaq Universal SYBR Green Supermix (Bio-Rad), on a QuantStudio6 according to manufacturer’s protocol. *IT4var20* transcription was quantified with specific primers [45] and delta CT (dCT) values were calculated relative to housekeeping gene *seryl-tRNA synthetase* (PF3D7_0717700) transcription quantified with established primers [20]. Transcript units (Tu) were calculated as Tu = 2^(5-ΔCtvar_gene)^ [18]. Primers were validated on tenfold genomic DNA dilutions to ensure correct amplification.

### RNA sequencing

Total RNA was prepared as libraries with the Kapa Stranded mRNA-seq library prep kit. Libraries from the two time series experiments (n = 64/experiment) were each sequenced on a full flow cell on the NovaSeq 6000 S2 platform with 150 base pair (bp) paired-end reads. Samples from Mali patients (n = 32) were sequenced on a full flow cell of the NovaSeq 6000 S1 platform with 50bp paired-end reads.

### RNA sequencing analyses

Reads were processed according to the methods as described in (Saelens et al. 2021, *Manuscript in preparation*). Briefly, reads were trimmed of adapter sequences and quality-filtered with Trimmomatic [46]. Human reads were depleted, and parasite reads were saved in new fastq files and quantified with Salmon [47] using the *P*. *falciparum* 3D7 transcriptome, assembly version ASM276.2. Quantification files of parasite gene expression were imported and summarized with the *tximport* package in R, and normalized abundance values of the *var* gene read counts were expressed as transcripts per million (TPM) [48] relative to the total read count for each sample.

Quantification of *var* gene transcripts from Mali patient samples was performed with fastq files depleted of human reads. These data were aligned to the 3D7 reference genome (assembly version GCA_000002765.3) with STAR. Reads that mapped to *var* genes were extracted with samtools [49] by specifying regions in a BED file of *var* gene loci in the 3D7 genome. A transcriptome file was created for *var* gene domains extracted from annotated *var* gene domains previously [11,15]. Reads were quantified with Salmon using a reference transcriptome index of k-mer size of 15. Quantification files of *var* gene domain expression were imported and summarized with the *tximport* package in R as unnormalized read counts. The unnormalized read counts were aggregated according to *var* gene domain type for each sample. Overall *var* gene transcription was assessed by the read counts of ATS domains. Aggregated read counts of other domains were normalized relative to the ATS domain read count for each sample.

### Adhesion assays

Petri-dish spot assays for recombinant proteins CD36 and EPCR were done as previously described [50], with following modifications. We coated 16 circled spots per falcon plate with 20 μl recombinant proteins in PBS overnight at 4°C in a humidity chamber. Two-fold protein concentration dilutions were prepared from 10 μg/ml to 0.31 μg/ml. Protein spots were blocked with Albumax at room temperature for 1 hour. Trophozoite stage iRBCs were purified with Percoll and adjusted to 4x10^6^ cells per ml. We added 20 μl iRBCs in Albumax complete media to each spot and incubated for 1 hour at 37°C in a humidity chamber. Petri-dishes were washed with complete media and PBS on a nutator and then fixed with 1.5% glutaraldehyde. Fixed spots were stained with 5% Giemsa for 20 min. Spots were imaged using a Zeiss Axio Imager A1 fluorescence microscope equipped with an Axio-Cam MRM digital and a 10X objective. From each spot, we used a consistent sampling scheme to obtain 3 images. From these, adhering parasites were quantified using ImageJ software (Fiji) [51] and R (version 4.0.0). Adhesion to each spot was normalized to the mean adhesion of parasites in normal blood to either CD36 or EPCR at 10 μg/ml for each replicate. A four-parameter log-logistic curve was fitted to the adhesion across protein spot concentrations for infected HbAA and HbAS RBCs separately using the drc package [52] in R (version 4.0.0).

### Flow cytometry

Late-stage iRBCs were purified by Percoll and split into HbAA and HbAS RBCs. After invasion, parasites were matured to trophozoite stage and then adjusted to 2 million cells per ml (0.5% hematocrit and 50 μl cells). We detected PfEMP1 using a primary monoclonal anti-IT4var20 PfEMP1 antibody at 100 μg/ml in complete medium for 30 min at 4°C, and goat anti-mouse APC-conjugated antibody (ThermoFischer) as secondary antibody. DNA in infected RBCs was stained with 40 μg/ml ethidium bromide. The cells were fixed in 1% formaldehyde in PBS, run on BD LSRFortessa flow cytometer. Gating and analysis and plots were created with Flowing software 2.5.1 (Cell Imaging and Cytometry, Turku Bioscience Centre) and R (version 4.0.0) with the FlowCore, Flowviz, FlowStats, and ggcyto packages [53–56].

## Supporting information

S1 Fig *Var* gene domain transcription. **A)** Normalized read counts of DBL domains. **B)** Normalized read counts of CIDR domains. Each dot represents the summarized count of the domain type in an individual. Read counts were normalized to ATS domain read counts for each sample. Dot and boxplots are colored according to hemoglobin genotype. Boxplots show median, interquartile range (IQR), and whiskers show 1.5 times the IQR.
(TIF)

S2 Fig Normalized expression of N-terminal segments. **A)** Normalized read counts for NTS and DBL domains according to associated types within each patient. Read counts were normalized to ATS domain read counts for each patient. A linear model trendline is marked in blue. **B)** Proportion of read counts for N-terminal segments (NTS) A, NTSB, and NTSpam normalized to ATS read counts in individual patient samples. Colors correspond to head structure type.
(TIF)

S1 Table Summary of raw read counts for the N-terminal domain types by hemoglobin genotype. Difference in aggregated domain read counts was calculated for the matched pairs of HbAA and HbAS samples. Mean, Standard deviation (SD), and min-max range listed.
(DOCX)
