# Supplementary figures and images for: Sickle-trait hemoglobin reduces adhesion to both CD36 and EPCR by Plasmodium falciparum-infected erythrocytes
Source: PLoS Pathog. 2021 Jun 11;17(6):e1009659. doi: 10.1371/journal.ppat.1009659 (PMC8221791; doi:10.1371/journal.ppat.1009659)

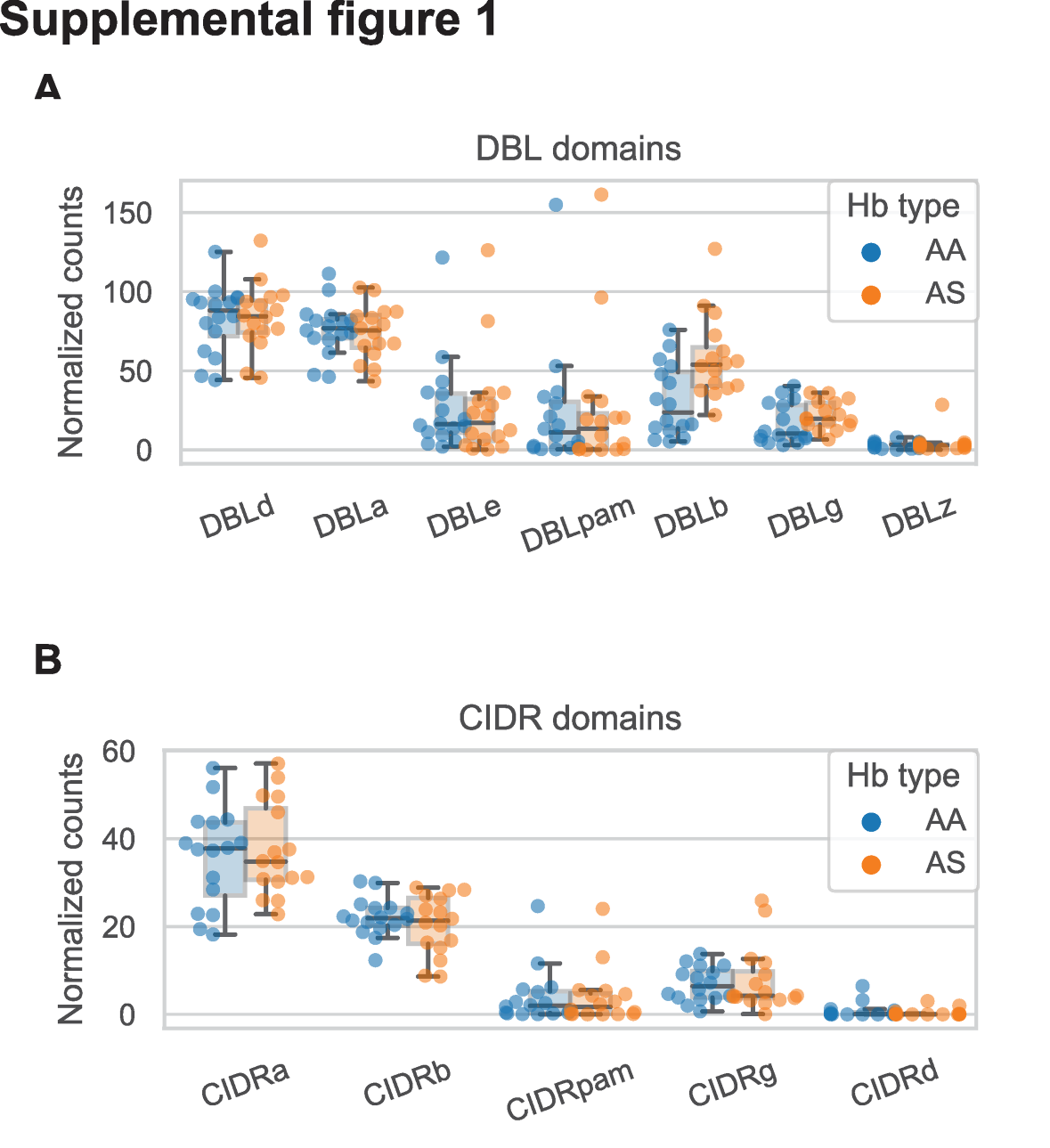

Supplement: S1 Fig — A) Normalized read counts of DBL domains. B) Normalized read counts of CIDR domains. Each dot represents the summarized count of the domain type in an individual. Read counts were normalized to ATS domain read counts for each sample. Dot and boxplots are colored according to hemoglobin genotype. Boxplots show median, interquartile range (IQR), and whiskers show 1.5 times the IQR. (TIF) [file ppat.1009659.s001.tif]

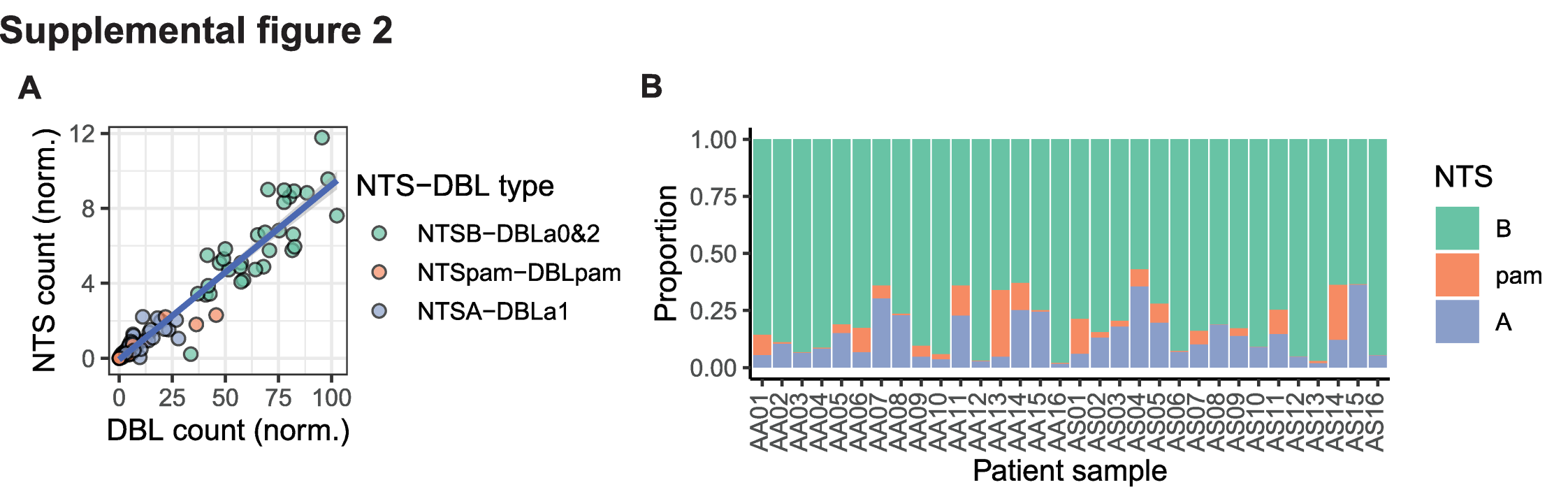

Supplement: S2 Fig — A) Normalized read counts for NTS and DBL domains according to associated types within each patient. Read counts were normalized to ATS domain read counts for each patient. A linear model trendline is marked in blue. B) Proportion of read counts for N-terminal segments (NTS) A, NTSB, and NTSpam normalized to ATS read counts in individual patient samples. Colors correspond to head structure type. (TIF) [file ppat.1009659.s002.tif]
