# Supplementary material for: Sickle-trait hemoglobin reduces adhesion to both CD36 and EPCR by Plasmodium falciparum-infected erythrocytes
Source: PLoS Pathog. 2021 Jun 11;17(6):e1009659. doi: 10.1371/journal.ppat.1009659 (PMC8221791; doi:10.1371/journal.ppat.1009659)
Supplement: S1 Table — Difference in aggregated domain read counts was calculated for the matched pairs of HbAA and HbAS samples. Mean, Standard deviation (SD), and min-max range listed. (DOCX) [file ppat.1009659.s003.docx]

Supplemental table 1. Domain raw read counts

|  | AA (N=16) | AS (N=16) | Difference (N=16) | p value |
| --- | --- | --- | --- | --- |
| **CIDRα1.2-7** |  |  |  | 0.159^1^ |
| Mean (SD) | 538.3 (626.6) | 956.8 (1039.3) | 418.5 (1093.5) |  |
| Range | 21.0 - 2574.1 | 48.0 - 3813.0 | -1214.9 - 3662.0 |  |
| **CIDRα1.1/8** |  |  |  | 1.000^1^ |
| Mean (SD) | 695.9 (1165.2) | 725.6 (951.1) | 29.8 (1045.6) |  |
| Range | 0.0 - 4247.7 | 3.0 - 2953.0 | -1514.0 - 2544.0 |  |
| **CIDRα2-6** |  |  |  | 0.860^1^ |
| Mean (SD) | 11990.4 (12003.3) | 14889.4 (22763.7) | 2899.0 (21738.4) |  |
| Range | 362.0 - 43100.3 | 223.8 - 97672.0 | -30603.4 - 72202.0 |  |
| **CIDRpam** |  |  |  | 0.821^1^ |
| Mean (SD) | 664.7 (823.3) | 934.6 (1355.1) | 269.8 (1283.2) |  |
| Range | 21.0 - 2867.0 | 2.0 - 4282.0 | -1573.0 - 3628.0 |  |
| **DBLα0** |  |  |  | 0.940^1^ |
| Mean (SD) | 26398.9 (28888.2) | 25003.7 (28821.7) | -1395.2 (20725.3) |  |
| Range | 966.8 - 98659.1 | 2743.9 - 124830.5 | -57280.1 - 26171.3 |  |
| **DBLα1** |  |  |  | 0.274^1^ |
| Mean (SD) | 2601.9 (2932.2) | 3675.1 (2796.9) | 1073.3 (4021.0) |  |
| Range | 68.8 - 10835.7 | 258.5 - 9257.9 | -8950.6 - 6292.7 |  |
| **DBLα2** |  |  |  | 0.433^1^ |
| Mean (SD) | 795.7 (977.1) | 851.6 (765.8) | 55.9 (768.7) |  |
| Range | 17.7 - 2967.3 | 79.6 - 2683.0 | -2149.6 - 1087.1 |  |
| **DBLpam** |  |  |  | 0.464^1^ |
| Mean (SD) | 912.6 (990.9) | 1524.9 (2239.5) | 612.3 (2135.2) |  |
| Range | 35.0 - 3023.0 | 8.0 - 8127.0 | -2218.2 - 7096.0 |  |

1. Wilcoxon signed rank exact test

Summary of raw read counts for the N-terminal domain types by hemoglobin genotype. Difference in aggregated domain read counts was calculated for the matched pairs of HbAA and HbAS samples. Mean, Standard deviation (SD), and min-max range listed.
